# Sex-based Disparities in Liver Transplantation for Hepatocellular Carcinoma and the Impact of the Growing Burden of NASH

**DOI:** 10.1097/TXD.0000000000001642

**Published:** 2024-06-20

**Authors:** Jia Hong Koh, Douglas Chee, Cheng Han Ng, Karn Wijarnpreecha, Mark Muthiah, Darren Jun Hao Tan, Wen Hui Lim, Rebecca Wenling Zeng, Benjamin Koh, Eunice Tan Xiang Xuan, Glenn Bonney, Shridhar Iyer, Dan Yock Young, Toru Nakamura, Hirokazu Takahashi, Mazen Noureddin, Mohammad Shadab Siddiqui, Tracey G. Simon, Rohit Loomba, Daniel Q. Huang

**Affiliations:** 1 Division of Gastroenterology and Hepatology, Department of Medicine, National University Hospital, Singapore, Singapore.; 2 Division of Gastroenterology and Hepatology, University of Arizona College of Medicine, Phoenix, AZ.; 3 MBBS Programme, Yong Loo Lin School of Medicine, National University of Singapore, Singapore, Singapore.; 4 Department of Surgery, National University Hospital, Singapore, Singapore.; 5 Division of Gastroenterology, Department of Medicine, Kurume University School of Medicine, Kurume City, Fukuoka, Japan.; 6 Liver Center, Saga University Hospital, Saga, Japan.; 7 Houston Liver Institute, Houston, TX.; 8 Division of Gastroenterology, Hepatology and Nutrition, Department of Internal Medicine, Virginia Commonwealth University, Richmond, VA.; 9 Division of Gastroenterology, Massachusetts General Hospital, Boston, MA.; 10 Division of Gastroenterology and Hepatology, Department of Medicine, University of California San Diego, La Jolla, CA.; 11 National University Centre for Organ Transplantation, National University Health System, Singapore.

## Abstract

**Background.:**

The cause of liver disease is changing, but its impact on liver transplantation (LT) for hepatocellular carcinoma (HCC) in women and men is unclear. We performed a nationwide study to assess the prevalence and posttransplant survival outcomes of the various causes of liver disease in women and men with HCC.

**Methods.:**

Data were obtained from the United Network for Organ Sharing database from 2000 to 2022. Data related to the listing, transplant, waitlist mortality, and posttransplant mortality for HCC were extracted. The proportion of HCC related to the various causes of liver disease among LT candidates and recipients and posttransplant survival were compared between women and men.

**Results.:**

A total of 51 721 individuals (39 465 men, 12 256 women) with HCC were included. From 2000 to 2022, nonalcoholic steatohepatitis (NASH) was the fastest-growing cause of liver disease among female LT candidates with HCC (*P* < 0.01), followed by alcohol-associated liver disease. NASH overtook chronic hepatitis C as the leading cause of liver disease in 2020 and 2022 among waitlisted women and men with HCC, respectively. Female patients with HCC spent a significantly longer time on the LT waitlist compared with male patients (β: 8.73; 95% confidence interval [CI], 2.91-14.54). Female patients with HCC from alcohol-associated liver disease also have a lower probability of receiving LT (subdistribution hazard ratio: 0.90; 95% CI, 0.82-0.99). Among transplant recipients with NASH HCC, female sex was associated with lower posttransplant mortality compared with male sex (hazard ratio: 0.79; 95% CI, 0.70-0.89; *P* < 0.01).

**Conclusions.:**

Women have a significantly longer waitlist duration compared with men. NASH is now the leading cause of liver disease among both female and male LT candidates and recipients with HCC.

Hepatocellular carcinoma (HCC) is the sixth most common cancer worldwide and the third leading cause of cancer-related mortality.^1^ Liver transplantation (LT) is the ideal curative therapy for people with HCC. However, it is associated with disparities in access, transplant receipt, and survival outcomes between men and women.^2-4^ Women are more likely to die on the LT waiting list and have a lower likelihood of receiving LT compared with men.^5,6^ However, it is unclear whether these sex-based disparities persist in LT candidates with HCC, given the unique selection criteria for candidates with HCC.^7^ In addition, the epidemiology of HCC is changing, with a rising burden of nonalcoholic steatohepatitis (NASH) and alcohol-related HCC.^8^

Emerging data suggest that mortality rates in female and male patients with NASH-related HCC are comparable, unlike other causes of liver disease,^9^ and NASH is now the leading cause of HCC in LT candidates.^10^ An updated study regarding sex-based disparities among waitlisted LT candidates with HCC has not been reported. It is unclear how the latest epidemiological shifts have impacted the comparative prevalence, transplant receipt, and posttransplant survival of female and male patients on the LT waitlist. Using registry data from the United Network for Organ Sharing database from 2000 to 2022, we performed a comprehensive, updated study to evaluate sex-based disparities in the prevalence, transplant receipt, and posttransplant survival of waitlisted LT candidates with HCC.

## MATERIALS AND METHODS

### Study Population

This study used data from the United Network for Organ Sharing Standard Transplant Analysis and Research database. The data included adult (aged 18 y or older) individuals with HCC who were on the LT waitlist or who had received an LT in the United States between January 1, 2000, and December 31, 2022. Patients listed before the introduction of model for end-stage liver disease (MELD) as the basis for LT allocation and those listed for multiorgan transplantation or liver retransplantation were excluded. Demographic data collected included age, sex, race/ethnicity, and body mass index (BMI). Obesity was defined as a BMI >30.0 kg/m^2^ for non-Asians and >27.5 kg/m^2^ for Asians. Race/ethnicity (eg, non-Hispanic White, Black, Hispanic, Asian) and sex were based on self-reported data. The following data were collected at the time of listing and at the time of transplantation: MELD score, serum creatinine, bilirubin, sodium, albumin, international normalized ratio, and tumor characteristics (eg, number of tumor nodules, maximum tumor size, maximum alpha-fetoprotein levels). Patients were considered to have HCC based on diagnosis codes. Both primary and secondary diagnostic codes were examined to classify patients by cause of liver disease. Patients with cryptogenic cirrhosis and a BMI ≥30.0 kg/m^2^ or diabetes mellitus were considered to have NASH.^11-14^ The outcomes of interest in this study were waitlist duration, receipt of LT, and post-LT mortality. All outcomes were censored at 10 y.

### Objectives

The primary objective was the proportion of HCC related to the various causes of liver disease among LT candidates, compared between women and men. Secondary objectives included the proportion of HCC related to the various causes of liver disease among LT recipients, the comparative duration on the waitlist, receipt of an LT, and posttransplant survival, compared between female and male candidates.

### Statistical Analysis

Categorical variables were presented as proportions with a 95% confidence interval (CI), whereas continuous variables were presented as medians with interquartile ranges [IQRs]. The Kruskal-Wallis test and the chi-square test were used to compare continuous variables and categorical variables. A linear-by-linear trend test was conducted with 10 000 Monte Carlo simulations to examine trends over time. Cox proportional analysis was used to compare overall posttransplant survival by sex and cause of liver disease. The analysis was adjusted for known confounders, including age, sex, race/ethnicity, diabetes mellitus, and MELD score at listing (model 1). An additional model (model 2) was constructed to include tumor-specific variables, such as tumor number, maximum alpha-fetoprotein levels, and largest tumor size. Waitlist duration among different causes of liver disease was assessed using quantile regression (to accommodate nonnormally distributed outcomes and allow for flexible modeling of nonlinear relationships with predictor variables),^15^ with clustering by study year and multivariable adjustment. The estimated regression coefficient indicated the difference in median waitlist duration between the independent variables, with a negative coefficient suggesting a shorter waitlist duration compared with the reference category (men). Competing risk regression using the Fine and Gray subdistribution hazard ratio (SHR) with multivariable adjustment was used to estimate the likelihood of receipt of LT with waitlist mortality as the competing event. Statistical analyses were performed using STATA (version 17.0), and a *P* value of ≤0.05 was considered statistically significant.

## RESULTS

### Characteristics of the Study Population

A total of 51 721 individuals with HCC listed for LT from 2000 to 2022 were included in this analysis. Of these patients, 39 465 were men and 12 256 were women. Six thousand four hundred fifty-seven of these patients had NASH, whereas 6030 had alcohol liver disease (ALD). Women had lower serum creatinine levels (0.79; IQR, 0.64–0.97) compared with men (0.90; IQR, 0.78–1.10) at the time of LT listing and had lower height (160.02 cm; IQR, 155.00–165.10 versus 175.26; IQR, 170.18–180.34) than men (Table 1). A summary of the clinical characteristics of individuals in each category is presented in **Tables S1 and S2** (**SDC,** http://links.lww.com/TXD/A667).

**TABLE 1.** Baseline characteristics of all liver transplant candidates with hepatocellular carcinoma 2000–2022

	Overall (men)	Overall (female)	*P*
Sample size	39 465	12 256	
At the time of liver transplant listing			
Age, y	60.00 (IQR, 55.00–65.00)	61.00 (IQR, 56.00–66.00)	<0.01
BMI, kg/m^2^	28.31 (IQR, 25.29–32.00)	28.31 (IQR, 24.43–32.95)	<0.01
Height, cm	175.26 (IQR, 170.18–180.34)	160.02 (IQR, 155.00–165.10)	<0.01
MELD score	11.00 (IQR, 8.00–14.00)	11.00 (IQR, 8.00–14.00)	<0.01
Albumin, g/dL	3.30 (IQR, 2.90–3.80)	3.20 (IQR, 2.80–3.70)	<0.01
INR	1.20 (IQR, 1.10–1.40)	1.20 (IQR, 1.10–1.40)	<0.01
Serum bilirubin, mg/dL	1.40 (IQR, 0.82–2.30)	1.40 (IQR, 0.90–2.40)	<0.01
Serum creatinine, mg/dL	0.90 (IQR, 0.78–1.10)	0.79 (IQR, 0.64–0.97)	<0.01
Serum sodium, mEq/L	138.00 (IQR, 135.00–140.00)	138.00 (IQR, 136.00–142.00)	<0.01
At the time of liver transplant surgery			
BMI, kg/m^2^	28.34 (IQR, 25.29–32.01)	28.32 (IQR, 24.42–32.95)	<0.01
MELD score	12.00 (IQR, 9.00–17.00)	12.00 (IQR, 9.00–18.00)	<0.01
Albumin, g/dL	3.30 (IQR, 2.80–3.80)	3.20 (IQR, 2.70–3.70)	<0.01
INR	1.30 (IQR, 1.10–1.58)	1.30 (IQR, 1.13–1.60)	<0.01
Serum bilirubin, mg/dL	1.60 (IQR, 0.90–3.00)	1.70 (IQR, 0.90–3.40)	<0.01
Serum creatinine, mg/dL	0.90 (IQR, 0.79–1.18)	0.80 (IQR, 0.66–1.07)	<0.01
Serum sodium, mEq/L	138.00 (IQR, 135.00–140.00)	138.00 (IQR, 135.00–140.00)	<0.01
Maximum tumor size, cm	2.80 (IQR, 2.00–4.00)	2.60 (IQR, 2.00–3.70)	<0.01
Tumor number	1.00 (IQR, 1.00–1.00)	1.00 (IQR, 1.00–1.00)	<0.01

BMI, body mass index; INR, international normalized ratio; IQR, interquartile range; MELD, model for end-stage liver disease.

### Trends in the Cause of Liver Disease Among LT Candidates With HCC by Sex

**FIGURE 1.**
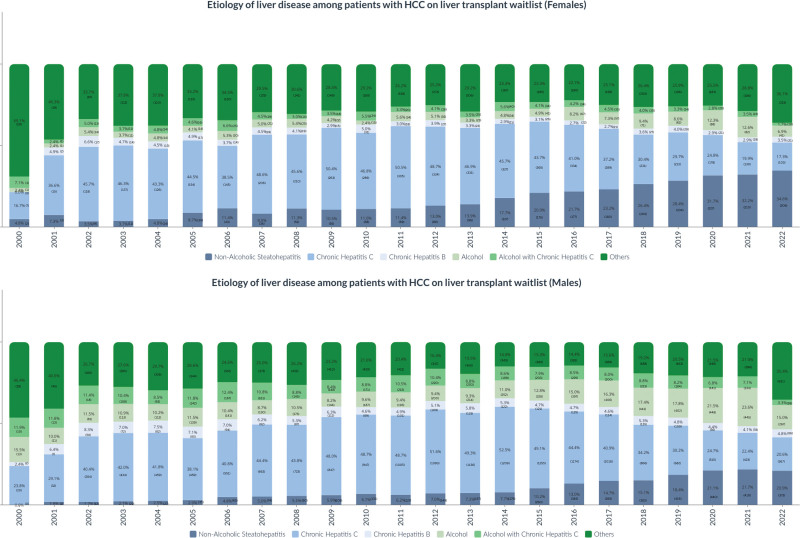
Cause of liver disease among patients with HCC on liver transplant waitlist. HCC, hepatocellular carcinoma.

In 2022, 596 female patients with HCC were on the waitlist for LT, compared with 1783 male patients. This represented a 1419% and 2123% increase in women and men, respectively, compared with 2000. Among women, NASH (*P* < 0.01) was the fastest-growing cause of liver disease patients with HCC on the waitlist, increasing from 4.76% in 2000 to 34.56% in 2022, followed by ALD (*P* < 0.01) increasing from 2.38% in 2000 to 6.88% in 2022 (Figure 1). By contrast, the proportion of waitlisted HCC patients with ALD with chronic hepatitis C (CHC) and other causes declined (*P* < 0.01). In 2020, NASH overtook CHC as the leading cause of liver disease among female patients with HCC waitlisted for LT (Figure 1).

Among men, the proportion of patients with NASH among waitlisted patients with HCC increased from 2000 to 2022 (*P* < 0.01; Figure 1). By contrast, the proportion of waitlisted male patients with CHC (*P* < 0.01), CHB (*P* < 0.01), alcohol (*P* < 0.01), ALD with CHC (*P* < 0.01), and other causes (*P* < 0.01) declined. The number of male patients on the waitlist with NASH increased from 0% in 2000 to 20.9% in 2022. In 2022, NASH overtook CHC as the leading cause of liver disease among male patients with HCC waitlisted for LT (Figure 1).

### Impact of Sex on LT Waitlist Duration

After adjusting for age, sex, height, race/ethnicity, history of diabetes mellitus, and MELD score, female patients with HCC had a significantly longer LT waitlist duration compared with male patients (β: 8.73; 95% CI, 2.91-14.54; Table 2). In model 2, additional adjustment for number of tumors, tumor size, and alpha-fetoprotein levels similarly found a significantly longer LT waitlist for female patients (β: 15.62; 95% CI, 12.74-18.50). There were similar findings in subgroup analyses of female patients with HCC related to NASH in model 2 (β: 9.28; 95% CI, 2.03-16.53).

**TABLE 2.** Impact of patient’s sex on waitlist duration, liver transplant receipt, and overall mortality after liver transplant

Liver transplant waitlist duration	Model 1	*P*	Model 2	*P*
Female (overall)	β: 8.73 (95% CI, 2.91 to 14.54)	**0.003**	β: 15.62 (95% CI, 12.74 to 18.50)	**<0.001**
Female (NASH)	β: 6.03 (95% CI, –5.50 to 17.57)	0.305	β: 9.28 (95% CI, 2.03 to 16.53)	**0.012**
Female (ALD)	β: 3.79 (95% CI, –13.44 to 21.03)	0.666	β: 10.11 (95% CI, –2.70 to 22.93)	0.122
**Liver transplant receipt**	**Model 1**	***P***	**Model 2**	***P***
Female (overall)	SHR: 0.97 (95% CI, 0.93 to 1.02)	0.327	SHR: 0.93 (95% CI, 0.87 to 0.98)	**0.015**
Female (NASH)	SHR: 0.96 (95% CI, 0.87 to 1.06)	0.380	SHR: 0.95 (95% CI, 0.87 to 1.03)	0.176
Female (ALD)	SHR: 0.90 (95% CI, 0.82 to 0.99)	**0.033**	SHR: 0.86 (95% CI, 0.77 to 0.98)	**0.018**
**Overall mortality after liver transplant**	**Model 3**	***P***	**Model 4**	***P***
Female (overall)	HR: 0.89 (95% CI, 0.79-1.01)	0.07	HR: 0.91 (95% CI, 0.79-1.05)	0.22
Female (NASH)	HR: 0.80 (95% CI, 0.71-0.91)	<0.01	HR: 0.79 (95% CI, 0.70-0.89)	<0.01
Female (ALD)	HR: 0.89 (95% CI, 0.71-1.11)	0.30	HR: 0.95 (95% CI, 0.73-1.25)	0.73

Model 1, adjusted for age, sex, height, ethnicity, personal history of diabetes mellitus, and MELD score. Model 2, adjusted for age, sex, height, ethnicity, personal history of diabetes mellitus, MELD score, number of tumors, tumor size, and alpha-fetoprotein levels.

Bolded *P* values ≤0.05 denote statistical significance.

ALD, alcohol liver disease; CI, confidence interval; MELD, model for end-stage liver disease; NASH, nonalcoholic steatohepatitis; REF, reference value; SHR, subdistribution hazard ratio.

### Impact of Sex on LT Receipt

Women had a lower overall likelihood of LT receipt in model 2 (SHR: 0.93; 95% CI, 0.87-0.98), with similar findings in sensitivity analyses for ALD (SHR: 0.86; 95% CI, 0.77-0.98), compared with men, after adjusting for age, sex, height, race/ethnicity, history of diabetes mellitus, MELD score, number of tumors, tumor size, and alpha-fetoprotein levels.

### Trends in the Cause of Liver Disease Among LT Recipients With HCC by Sex

**FIGURE 2.**
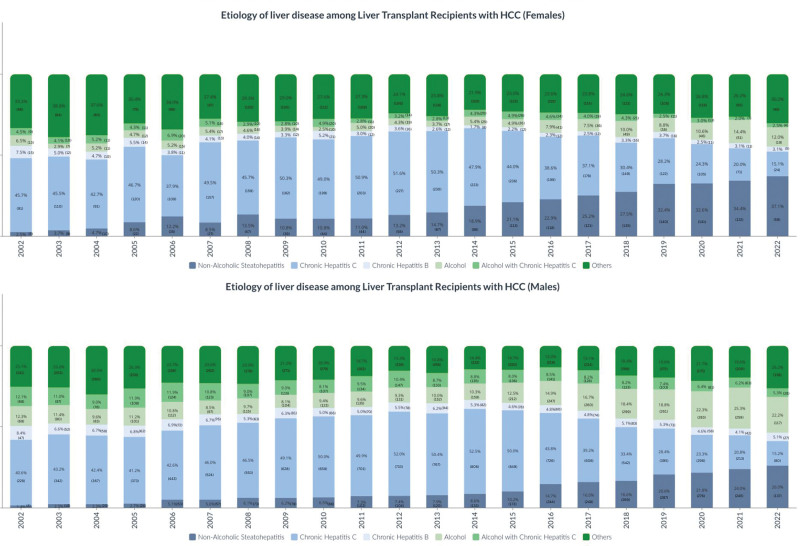
Cause of liver disease among liver transplant recipients with HCC. HCC, hepatocellular carcinoma.

**FIGURE 3.**
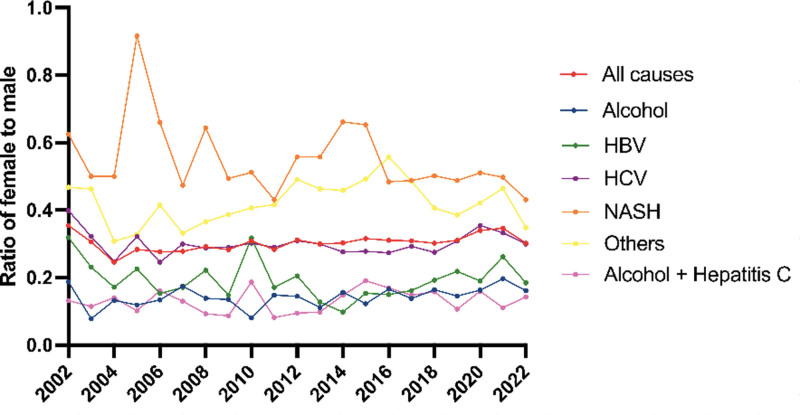
Female-to-male ratio of liver transplant recipients with HCC. HBV, hepatitis B virus; HCC, hepatocellular carcinoma; HCV, hepatitis C virus; NASH, nonalcoholic steatohepatitis.

In 2022, 159 female patients with HCC on the LT waitlist received a transplant, compared with 527 male patients. From 2002 to 2022, NASH was the fastest-growing cause of liver disease among female LT recipients with HCC (*P* < 0.01); 2.5% of LT recipients with HCC had NASH in 2002, compared with 37.1% of LT recipients in 2022 (Figure 2). This was followed by ALD, increasing from 6.5% in 2002 to 12.0% in 2022. By contrast, the proportion of HCC transplant recipients with CHC, CHB, ALD with CHC, and other causes declined (*P* < 0.01). NASH surpassed CHC to become the leading cause of liver disease among female LT recipients with HCC in 2019. Among male LT recipients with HCC, NASH was also the fastest-growing cause of liver disease (*P* < 0.01), increasing from 1.4% in 2002 to 26.0% in 2022 (Figure 2). This was followed by ALD, increasing from 12.3% in 2002 to 22.2% in 2022. The overall female-to-male ratio of LT recipients with HCC was 0.35 in 2002 and 0.30 in 2022 (Figure 3). By cause of liver disease, in 2022, the female-to-male ratio ranged from 0.43 in NASH to 0.14 in ALD.

### Posttransplant Mortality

Among transplant recipients with HCC, female sex had similar posttransplant mortality compared with male sex (model 3: HR: 0.89; 95% CI, 0.79-1.01; *P* = 0.07), after adjustment for age, sex, race/ethnicity, diabetes mellitus, and MELD score. These findings remained consistent in a separate multivariable model (model 4: HR: 0.91; 95% CI, 0.79-1.05; *P* = 0.22), which adjusted for tumor-specific factors, in addition to the covariates included in model 3. However, in sensitivity analysis for NASH, women had a lower risk of posttransplant mortality in both model 3 (HR: 0.80; 95% CI, 0.71-0.91; *P* < 0.01) and model 4 (HR: 0.79; 95% CI, 0.70-0.89; *P* < 0.01). The impact of sex on LT waitlist duration, LT receipt, and overall mortality after LT is presented in detail in Table 2.

## DISCUSSION

### Main Findings

In this nationwide study of 51 721 individuals with HCC listed for LT in the United States, we determined that women spent a significantly longer duration on the LT waitlisted and had a lower likelihood of receiving a transplant compared with men. NASH is now the leading cause of liver disease among both female and male LT candidates and recipients with HCC. There was no significant difference in posttransplant mortality between female and male patients. However, female patients with NASH-related HCC had lower posttransplant mortality compared with male patients. The current study demonstrates the substantial disparities between female and male patients with HCC, and it adds to the growing body of literature showing that NASH is a rapidly growing cause of HCC in both female and male patients.^9,16^

### In Context With Current Literature

Our findings of sex-related disparities in LT among candidates with HCC add to the growing body of evidence regarding sex disparities for LT in general.^5,17^ Recent studies have demonstrated how sex-related disparities arose from the MELD score^18,19^ and related to height.^20,21^ Although the new MELD 3.0 score^6^ shows promise in bridging the gap in disparities between men and women in general on the LT waitlist, its impact on candidates with HCC is unclear.

### Strengths and Limitations

The strengths of our study include its large sample size, the inclusion of nationwide data, the inclusion of recent data up till 2022, and the adjustment for multiple confounders. Limitations include its retrospective design, the potential for residual confounding, and nonuniformity of the transplant criteria over the years (transition from Child-Turcotte-Pugh to MELD, then MELD-Na). We cannot confirm an equal distribution of exception points between male and female patients with HCC. In addition, further time points may have to be explored to better understand the clinical progression of patients with HCC over time while on the LT waitlist.

Our study also demonstrated lower posttransplant mortality in female patients with NASH-related HCC compared with male patients, and further studies will be needed to fully characterize the unique, sex-specific risk factors that influence post-LT mortality.

### Implications for Further Research and Clinical Practice

This study highlights the immense burden that NASH poses on the healthcare system. With NASH now the leading cause of liver disease among waitlisted female and male patients with HCC, there is an urgent need to improve preventive strategies to reduce the risk of HCC.^22,23^ This study serves as a call to action to increase research in preventive and surveillance strategies for NASH.^24,25^ The current study also highlights the challenges and disparities faced by female patients with HCC on the LT waitlist. Further studies are required to determine the reasons behind these disparities and to develop optimal allocation strategies for waitlisted female and male patients with HCC.

## CONCLUSION

Women spend a longer duration on the waitlist and are less likely to receive transplantation compared with men. NASH is now the leading cause of liver disease among both female and male waitlisted candidates and recipients with HCC. Female patients with HCC derive similar benefits from LT as male patients, and those with NASH-related HCC may have better post-LT outcomes compared with male patients. Further research is required to determine optimal organ allocation strategies for people with HCC to reduce these disparities.

## Supplementary Material


