# Supplementary material for: Sex-based Disparities in Liver Transplantation for Hepatocellular Carcinoma and the Impact of the Growing Burden of NASH
Source: Transplant Direct. 2024 Jun 20;10(7):e1642. doi: 10.1097/TXD.0000000000001642 (PMC11191941; doi:10.1097/TXD.0000000000001642)
Supplement: Supplementary file 1 [file txd-10-e1642-s001.pdf]

Table S1: Baseline Characteristics of NASH Liver Transplant Candidates with Hepatocellular Carcinoma 2000-2022

	**NASH (Male)**	**NASH (Female)**	**P-value**
Sample Size	4,174	2,283	
At the time of Liver Transplant Listing			
Age (years)	64.00 (IQR: 59.00 to 68.00)	65.00 (IQR: 60.00 to 68.00)	<0.01
BMI (kg/m2)	31.97 (IQR: 28.50 to 35.73)	32.11 (IQR: 28.29 to 36.08)	<0.01
Height (cm)	175.26 (IQR: 170.18 to 180.34)	160.00 (IQR: 154.94 to 165.10)	<0.01
MELD Score	12.00 (IQR: 9.00 to 16.00)	11.00 (IQR: 8.00 to 15.00)	<0.01
Albumin (g/dL)	3.30 (IQR: 2.90 to 3.80)	3.30 (IQR: 2.90 to 3.60)	<0.01
INR	1.30 (IQR: 1.10 to 1.40)	1.22 (IQR: 1.10 to 1.40)	<0.01
Serum Bilirubin (mg/dL)	1.60 (IQR: 1.00 to 2.50)	1.60 (IQR: 0.90 to 2.50)	<0.01
Serum Creatinine (mg/dL)	0.90 (IQR: 0.77 to 1.14)	0.80 (IQR: 0.63 to 1.00)	<0.01
Serum Sodium (mEq/L)	138.00 (IQR: 135.00 to 140.00)	138.00 (IQR: 135.00 to 140.00)	<0.01
At the time of Liver Transplant Surgery			
BMI (kg/m2)	31.95 (IQR: 28.48 to 35.63)	32.06 (IQR: 28.26 to 35.99)	<0.01
MELD Score	14.00 (IQR: 10.00 to 20.00)	14.00 (IQR: 10.00 to 21.00)	<0.01
Albumin (g/dL)	3.20 (IQR: 2.80 to 3.70)	3.20 (IQR: 2.70 to 3.60)	<0.01
INR	1.32 (IQR: 1.20 to 1.70)	1.30 (IQR: 1.20 to 1.70)	<0.01
Serum Bilirubin (mg/dL)	1.90 (IQR: 1.10 to 3.50)	2.00 (IQR: 1.10 to 3.80)	<0.01

Serum Creatinine (mg/dL)	0.99 (IQR: 0.79 to 1.30)	0.84 (IQR: 0.68 to 1.18)	<0.01
Serum Sodium (mEq/L)	137.00 (IQR: 134.00 to 140.00)	138.00 (IQR: 135.00 to 140.00)	<0.01
Maximum Tumour Size (cm)	2.60 (IQR: 2.00 to 3.90)	2.50 (IQR: 2.00 to 3.60)	<0.01
Tumor Number	1.00 (IQR: 1.00 to 1.00)	1.00 (IQR: 1.00 to 1.00)	<0.01

Legend: NASH, Non-alcoholic Steatohepatitis; IQR, Interquartile Range; BMI, Body Mass Index; MELD, Model for End-Stage Liver Disease; INR, International Normalised Ratio;

Table S2: Baseline Characteristics of Alcohol Liver Transplant Candidates with Hepatocellular Carcinoma 2000-2022

	**Alcohol (Male)**	**Alcohol (Female)**	**P-value**
Sample Size	5,237	793	
At the time of Liver Transplant Listing			
Age (years)	61.00 (IQR: 56.00 to 66.00)	57.00 (IQR: 50.00 to 63.00)	<0.01
BMI (kg/m2)	29.07 (IQR: 26.13 to 32.55)	26.27 (IQR: 23.04 to 30.62)	<0.01
Height (cm)	175.26 (IQR: 170.18 to 180.34)	162.56 (IQR: 157.48 to 167.64)	<0.01
MELD Score	13.00 (IQR: 10.00 to 17.00)	14.00 (IQR: 10.00 to 19.00)	<0.01
Albumin (g/dL)	3.20 (IQR: 2.80 to 3.60)	3.20 (IQR: 2.80 to 3.70)	<0.01
INR	1.30 (IQR: 1.20 to 1.50)	1.30 (IQR: 1.20 to 1.60)	<0.01
Serum Bilirubin (mg/dL)	1.80 (IQR: 1.10 to 3.10)	2.20 (IQR: 1.20 to 4.00)	<0.01
Serum Creatinine (mg/dL)	0.90 (IQR: 0.77 to 1.11)	0.83 (IQR: 0.68 to 1.07)	<0.01
Serum Sodium (mEq/L)	137.00 (IQR: 134.00 to 141.00)	137.00 (IQR: 134.00 to 139.00)	<0.01
At the time of Liver Transplant Surgery			
BMI (kg/m2)	29.12 (IQR: 26.15 to 32.55)	26.32 (IQR: 23.03 to 30.68)	<0.01
MELD Score	14.00 (IQR: 10.00 to 21.00)	15.00 (IQR: 10.00 to 22.00)	<0.01
Albumin (g/dL)	3.20 (IQR: 2.70 to 3.60)	3.20 (IQR: 2.80 to 3.70)	<0.01
INR	1.40 (IQR: 1.20 to 1.70)	1.40 (IQR: 1.20 to 1.80)	<0.01
Serum Bilirubin (mg/dL)	2.10 (IQR: 1.20 to 3.90)	2.30 (IQR: 1.30 to 4.70)	<0.01

Serum Creatinine (mg/dL)	0.94 (IQR: 0.79 to 1.24)	0.86 (IQR: 0.70 to 1.10)	<0.01
Serum Sodium (mEq/L)	137.00 (IQR: 134.00 to 140.00)	137.00 (IQR: 134.00 to 140.00)	<0.01
Maximum Tumour Size (cm)	2.7 (IQR: 2.00 to 3.90)	2.50 (IQR: 2.00 to 3.60)	<0.01
Tumor Number	1.00 (IQR: 1.00 to 1.00)	1.00 (IQR: 1.00 to 1.00)	<0.01

Legend: IQR, Interquartile Range; BMI, Body Mass Index; MELD, Model for End-Stage Liver Disease; INR, International Normalised Ratio;
